# Early Major Adverse Cardiovascular Event in a Patient With Chronic Kidney Disease: A Case Report

**DOI:** 10.7759/cureus.40161

**Published:** 2023-06-08

**Authors:** Saleh Saleh Juman Alseiari, Reem Fadil Ali Al Thehli, Munawar Farooq

**Affiliations:** 1 Department of Emergency Medicine, College of Medicine and Health Sciences, United Arab Emirates University, Al Ain, ARE; 2 Department of Internal Medicine, Emergency Medicine Section, College of Medicine and Health Sciences, United Arab Emirates University, Al Ain, ARE

**Keywords:** acute heart failure, major adverse cardiovascular event, chest pain, myocardial infarction, cardiac biomarkers, troponin rise, mace, acute coronary syndromes, acs, chronic kidney disease

## Abstract

Acute coronary syndrome (ACS) is common in people with chronic kidney disease (CKD) and is linked to poor short- and long-term outcomes. The diagnosis of myocardial infarction is challenging in patients with CKD as they have baseline elevated troponin levels. To date, there are no widely accepted guidelines to suggest what is a clinically significant change in troponin levels in these patients. We report a case of a patient with CKD who presented with chest pain to the emergency department (ED). His baseline troponin was high; however, the delta change was 11%. He was discharged from the ED for outpatient follow-up, but within 36 hours, he had significant ST elevation myocardial infarction (STEMI) with unstable hemodynamics and acute heart failure requiring urgent intubation and coronary revascularization. This case highlights the gap in clinical knowledge and practice in a relatively not uncommon presentation in emergency departments.

## Introduction

Acute coronary syndrome (ACS) is a time-critical condition that requires prompt management to ensure good outcomes. The diagnosis of ACS is made through clinical presentation, electrocardiogram (ECG), and/or biomarkers. The major goals of evaluation of patients with chest pain in the emergency department are to risk stratify, offer timely care, and avoid major adverse cardiovascular events (MACEs) in patients after discharge from the hospital. In patients with advanced chronic kidney disease (CKD), it is especially challenging to diagnose ACS as they have a baseline elevated troponin level because of varied mechanisms [1]. One commonly proposed mechanism is that there is a nonischemic myocardial injury occurring in patients with CKD [2]. CKD is an important risk factor that increases the risk of premature cardiovascular disease by 25%-30% and doubles the mortality rate of ACS [3]. Since specific criteria/guidelines do not exist to help exclude an ACS in patients with CKD, many cases are missed or are diagnosed late, which can lead to potentially preventable complications. Here, we present a case report of a male patient with stage 5 CKD who developed ST elevation myocardial infarction (STEMI) and acute heart failure requiring intubation and coronary revascularization within 36 hours after his first presentation to the emergency department (ED) with chest pain.

## Case presentation

A 67-year-old male with a known history of stage 5 CKD, type 2 diabetes mellitus (DM), hypertension, and hyperlipidemia presented to the ED with a history of left-sided heavy pressure-like chest pain over the last several hours, associated with mild shortness of breath. The severity of pain was 5 out of 10 with no radiation. The patient experienced similar pain over the last 10 days, but it was relatively more severe and constant on the day of presentation. There was no significant association with exertion, and there were no relieving or aggravating factors. Although the patient had symptoms for 10 days, he presented to the hospital as he was more concerned about the associated shortness of breath.

The patient had a history of two percutaneous coronary interventions (PCIs) first in 2007 and second in 2014. He was a smoker with 40 pack-years of smoking. The patient was regularly taking tablet aspirin and antihypertensive medications and had never been on dialysis.

On examination, he looked well, with normal vital signs and normal oxygen saturations. Examination of the chest was unremarkable with normal bilateral air entry with no added sounds. The patient was given tablet aspirin 300 mg soon after arrival in the ED.

**Figure 1:**
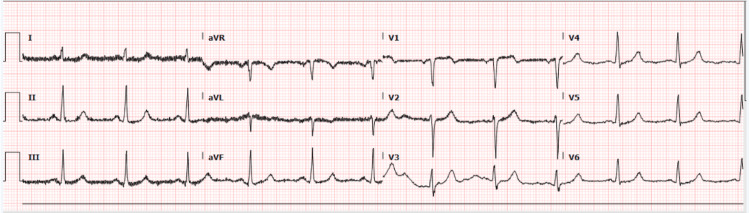
ECG on the first presentation ECG: electrocardiogram

His ECG showed sinus rhythm with no acute coronary ischemic changes (Figure 1), and his first troponin T was 104 ng/L (normal value: <14 ng/L). His serum creatinine was 507 μmol/L. There was no previous baseline troponin level available for the patient. Because of the high index of clinical suspicion, the cardiology team was consulted. The patient's symptoms had resolved completely. Because he had stage 5 CKD, a serial troponin was advised in three hours to see if it was static or increasing. The second troponin was 116 ng/L (an 11% increase from the first troponin).

It was implied that the delta change of 11% was insignificant given the patient's creatinine level of 507 μmol/L. It was also considered that it is atypical for the patient to have similar chest pain for 10 days. Also, a normal ECG and the fact that the patient's symptoms had resolved completely were reassuring for the treating team. The cardiology and emergency teams discussed the disposition options with the patient. The patient was offered admission but was also advised that if he wanted to go home, that was equally fine. The patient was discharged by the ED and cardiology team as per the patient's preference. Outpatient referrals to renal and cardiology clinics were advised.

**Figure 2:**
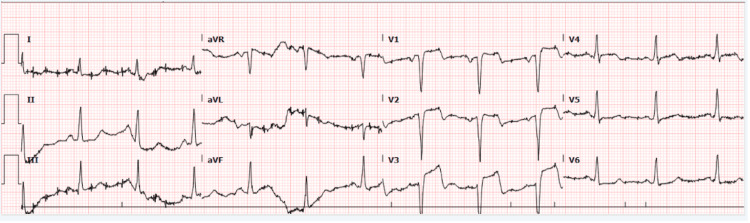
ECG on the second presentation Baseline wander in ECG as the patient had respiratory distress due to acute heart failure with new ST elevation in V1-3 ECG: electrocardiogram

After 36 hours, he re-presented with severe chest pain that started four hours before attending the hospital. He described the pain as severe and pressure-like that it radiated to his left arm and was associated with shortness of breath. This time, the patient was in distress with abnormal vital signs. He was hypertensive and tachypneic, with saturations of 88%-92% on room air (Table 1). He was orthopneic due to florid pulmonary edema and rapidly progressed to respiratory failure warranting endotracheal intubation. ECG had baseline wander because of respiratory distress, but there was clear ST elevation in leads V1-3, consistent with anteroseptal STEMI (Figure 2).

**Table 1:** Clinical parameters in the first and second presentations within 48 hours SOB: shortness of breath, R/R: respiratory rate, BP: blood pressure, ED: emergency department, ICU: intensive care unit, ECG: electrocardiogram

Clinical parameter	First presentation	Second presentation
Chest pain	Left-sided pressure-like chest pain, intermittent for 10 days, for a few hours before presentation, constant and severe, some feeling of SOB, no radiation	Similar pain as in the first presentation for four hours, this time also radiating to the left arm and SOB was more marked
General appearance	Appeared well, in discomfort	Unwell with respiratory distress and chest pain
Vital signs	Pulse: 70/minute, R/R: 17/minute, BP: 147/71 mmHg, oxygen saturation: 100% on room air	Pulse: 72/minute, R/R: 22/minute, BP: 153/73 mmHg, oxygen saturation: 88%-92% on room air
Chest auscultation	Bilateral clear breath sounds	Widespread bilateral crepitations
ECG	No ST elevation	ST elevation V1-3
Serum creatinine	507 μmol/L	497 μmol/L
Troponin T levels	104 and 116 ng/L	161 ng/L
Length of stay	Five hours in the ED	Three days in the ICU, 11 days in the cardiology ward

The catheterization laboratory was activated. The troponin result came back when the patient was still in the ED for stabilization. Troponin T was 161 ng/L. On angiogram, there was 99% ostio-proximal severe hazy stenosis with thrombus in the left anterior descending (LAD) coronary artery. The patient received percutaneous coronary intervention (PCI). He made a good recovery and was transferred to the ward from the ICU after three days (Table 1).

His predischarge echocardiogram revealed normal systolic and diastolic ventricular function indicating that acute heart failure was precipitated by ST elevation myocardial infarction and was reversed after the stent was placed in ostio-proximal LAD coronary artery.

## Discussion

The case report highlights difficulties in clinical decision-making in patients with advanced renal disease and ACS. In this patient, a coronary angiogram done on the second presentation revealed that the patient had 99% stenosis with a thrombus in the LAD, which was most likely the cause of his first presentation to the ED. The elevated troponin levels were disregarded due to CKD. He had a major adverse cardiovascular event (MACE) within 48 hours of discharge when he presented with acute ST elevation myocardial infarction and acute heart failure. On the first presentation, the repeat troponin level results for this patient showed less than 20% change, which led the treating team to think that it was a static rise in troponin and to discharge the patient. This shows the significance of the absence of a clear guideline to diagnose ACS in CKD patients in the presence of raised troponin T levels. The management of ACS depends on symptoms, ECG changes, cardiac enzyme changes, and risk stratification. This was a case of ACS in a patient with advanced renal disease, which was missed in the first presentation.

As it is difficult to rule out ACS in CKD patients, there are different approaches and recommendations that help diagnose ACS in this group of patients. One recommendation is to have serial troponin tests, and if it shows more than a 20% change, it can be considered positive [4,5]. It has been documented that CKD patients have more likelihood of an atypical presentation with the prevalence of typical chest pain among patients with ACS being inversely related to the stage of CKD [6]. CKD patients have variable ECG changes according to the severity of their renal dysfunction and associated electrolyte disturbances, with fewer STEMI, more non-ST-elevation myocardial infarction (NSTEMI), and left bundle branch block [6]. All these factors contribute to the challenging task of ruling out ACS in this group of patients.

Biomarkers have become an important diagnostic tool in emergency medicine and cardiology. Since it is known that the specificity of troponin levels is decreased in CKD patients, there is a need for other diagnostic and prognostic markers that are sensitive and specific even with advanced renal disease. There are biomarkers that are being tested to determine their specificity in diagnosing ACS in different groups of patients. Such markers include myeloperoxidase, matrix metalloproteinases (MMP-9), and CD40 ligand, which is a plaque rupture marker [1]. It still remains to be established if these newer biomarkers will also be useful in diagnosing ACS in patients with CKD. For now, the high index of suspicion needs to be maintained while risk stratifying these patients.

In a recent cluster-randomized controlled trial, a high-sensitivity troponin I (TnI) protocol was introduced in the diagnostic pathway of ACS [7]. It was found that although it increased the number of patients with advanced renal disease who were diagnosed with acute myocardial injury, it did not lead to an increased rate of coronary revascularization procedures and did not change any patient-related outcomes. Our case report also highlights the need for improvement in the diagnosis and management of patients with CKD presenting with chest pain.

## Conclusions

Cardiac troponins are the cornerstone in the evaluation of patients with chest pain, but it remains challenging to rule out ACS in patients with advanced renal disease. This case report is about a patient with CKD who had a high-risk clinical presentation for ACS, which was missed due to the following factors: the ECG was normal, the troponin rise was less than 20% (cutoff mentioned in some guidelines), and the patient's symptom of intermittent chest pain for 10 days was considered an atypical chest pain. The patient developed MACE in 36 hours, and coronary angiogram findings confirmed that his first presentation was due to ACS. For this relatively not uncommon and high-risk presentation, decision-making in the emergency room is variable and lacks guidelines based on robust evidence. Until we have clear evidence-based guidelines about interpretations of raised biomarkers in ACS in advanced CKD, it is imperative for physicians to use a broader evaluation criterion that should include risk factors, current presentation, ECG, and biomarker level to prevent an early MACE. This case report aims to put more emphasis on clinical presentation than on biomarkers in this high-risk population.
